# A 3D Composite Model Using Electrospinning Technology to Study Endothelial Damage

**DOI:** 10.3390/biom15060865

**Published:** 2025-06-13

**Authors:** Carmen Ciavarella, Luana Di Lisa, Gianandrea Pasquinelli, Maria Letizia Focarete, Sabrina Valente

**Affiliations:** 1 Department of Medical and Surgical Sciences (DIMEC), University of Bologna, 40138 Bologna, Italy; carmen.ciavarella2@unibo.it (C.C.); gianandr.pasquinelli@unibo.it (G.P.); 2 Department of Chemistry “Giacomo Ciamician”, University of Bologna, 40126 Bologna, Italy; luana.dilisa2@unibo.it (L.D.L.); marialetizia.focarete@unibo.it (M.L.F.); 3 IRCCS Azienda Ospedaliero-Universitaria di Bologna, 40138 Bologna, Italy

**Keywords:** electrospun scaffolds, endothelial dysfunction, oxidized LDL, shear stress, bioreactor

## Abstract

Background: Endothelial dysfunction triggers atherosclerosis pathogenesis. This study aimed at developing a 3D scaffold model able to reproduce in vitro the human vascular intima and study the endothelial damage induced by oxidative low-density lipoproteins (ox-LDLs) and shear stress. (2) Methods: Three-dimensional sandwich-like scaffolds were fabricated using electrospinning technology, functionalized with type I collagen and laminin, and subsequently coated with methacrylated gelatin hydrogel (GelMa) to achieve the final composite structure. Human umbilical vein endothelial cells (HUVECs) were used as the cell model for testing the suitability of 3D supports for cell culture exposed to ox-LDL both under static and shear stress conditions. Cell viability, ultrastructural morphology, and nitric oxide (NO) levels were analyzed. (3) Results: Electrospun mats and their functionalization were optimized to reproduce the chemical and physical properties of the vascular intima tunica. The 3D supports were suitable for the cell culture. Ox-LDL did not affect the HUVEC behavior in the 3D models under a static environment. Conversely, high shear stress (500 µL/min, HSS) significantly decreased the cell viability, also under the ox-LDL treatment. (4) Conclusions: Endothelial cell cultures on electrospun supports exposed to HSS provide a candidate in vitro model for investigating the endothelial dysfunction in atherosclerosis research. Technical improvements to the experimental setting are necessary for validating and standardizing the suggested 3D model.

## 1. Introduction

The human endothelium exerts a pivotal role in cardiovascular health and homeostasis. Indeed, it consists of a monolayer of endothelial cells (ECs), which line the internal surface of blood vessels and ensure vascular integrity, anti-platelet aggregation, and fibrinolytic properties [1]. Furthermore, ECs regulate vascular hemostasis and alleviate the inflammatory process. Most of these functions are mediated by nitric oxide (NO), which is an endogenous vasodilator [1]. Oxidative stress, inflammation, dyslipidemia, alterations in laminar blood flow, and shear stress are triggering factors in EC alterations leading to endothelial dysfunction [2]. This condition is characterized by a functional impairment of ECs and represents a critical step in atherosclerosis pathogenesis. The loss of NO promotes low-density lipoprotein (LDL) oxidation and internalization by smooth muscle cells (SMCs) and macrophages in the well-known foam cells, which form the initial stage of atherosclerotic plaque [3,4,5]. The inflammatory process exacerbation, vascular remodeling, and further complications, like plaque calcification, aggravate the atherosclerotic lesion, leading to high rupture risk. Considering the high incidence and mortality rate due to thromboembolic events, unveiling the main cellular and molecular mechanisms involved in atherosclerosis is crucial for novel and effective therapeutic strategies. Given the complexity of atherosclerosis, it is essential to develop advanced in vitro models that accurately replicate endothelial dysfunction to study disease mechanisms and explore novel therapeutic strategies.

Traditional in vitro studies rely on static 2D cell cultures, where ECs are grown as monolayers on plastic substrates [6,7]. However, these models present intrinsic limitations, as they fail to replicate the complex three-dimensional (3D) microenvironment of native tissues [8,9]. To address these limitations, various 3D models have been developed, including spheroids, microfluidic “vessel-on-a-chip” platforms [10], and engineered tissues, which better mimic cell–cell and cell–extracellular matrix (ECM) interactions [11,12,13]. Among these, hydrogel-based 3D models have emerged as promising platforms due to their ability to mimic the biochemical and mechanical properties of the ECM [14]. Gelatin methacrylate (GelMa), a chemically modified gelatin, is widely used in tissue engineering applications due to its biocompatibility, tunable mechanical properties, and ability to support cell adhesion and proliferation [15]. By adjusting the degree of methacrylation, key properties such as porosity, stiffness, and enzymatic degradability can be fine-tuned to meet specific target-tissue requirements [16,17]. Recent studies have leveraged these features to develop GelMa-based atherosclerosis models, such as two-layered GelMa hydrogel incorporating endothelial cells (ECs) and smooth muscle cells (SMCs), which has been used to investigate the role of zinc ions in atherosclerosis therapy [18].

Building on the advantages of hydrogel-based 3D models, electrospinning technology provides an effective method to fabricate scaffolds that resemble the fibrous architecture of the ECM [19,20]. For instance, Zhao et al. developed a PCL/fibrin vascular scaffold using electrospinning, which exhibited good biomechanical properties, cell compatibility, and degradation characteristics. In vivo studies in abdominal aorta models showed that the PCL/fibrin grafts mimicked native artery function, with improved ECM deposition, endothelialization, and reduced calcification compared to PCL alone [21]. In this study, we selected poly(L-lactide-co-ε-caprolactone) (PLCL) as the base polymer due to its biodegradability, mechanical resilience, and elastomeric properties, which are crucial for vascular tissue engineering applications [22]. PLCL exhibits tunable mechanical properties that can be modulated by altering the copolymer ratio, which allows for the replication of the native vascular tissues [23]. This mechanoelastic behavior supports endothelial cell proliferation under cyclic strain, which is relevant for mimicking the dynamic environment of blood vessels [24]. Furthermore, PLCL has been successfully used in various soft tissue regeneration applications, such as blood vessels, tendons, skin, and cardiac tissues, where flexibility is essential to withstand high blood pressure [25]. To enhance bioactivity, the electrospun PLCL mat was functionalized with type I collagen, a major ECM component that provides cell adhesion sites and biochemical cues essential for endothelial cell attachment and function [26].

This study aimed to reproduce an in vitro biomimetic model of the human tunica intima to investigate the endothelial dysfunction under atherosclerotic conditions. To achieve this, a multilayered 3D composite scaffold was engineered by integrating electrospun PLCL nanofibers with a GelMa hydrogel core, creating a physiologically relevant microenvironment that replicates the structural, biomechanical, and biochemical features of native vascular tissue. To mimic endothelial dysfunction observed in early-stage atherosclerosis, human umbilical vein endothelial cells (HUVECs) were seeded onto the composite scaffold and exposed to oxidized low-density lipoproteins (ox-LDLs) and shear stress variations [27]. Shear stress, a key biomechanical factor regulating endothelial homeostasis, plays a pivotal role in atherosclerosis progression. Low and oscillatory shear stress patterns have been associated with pro-inflammatory endothelial phenotypes, favoring lipid accumulation and plaque formation, while high laminar shear stress exerts protective effects on vascular function [28,29]. The multilayered electrospun PLCL-GelMa-PLCL composite scaffold represents an innovative approach for vascular tissue engineering, providing a biomimetic platform that closely resembles the human tunica intima. This model offers a promising tool for in vitro disease modeling, enabling the study of endothelial dysfunction under physiologically relevant conditions.

## 2. Materials and Methods

### 2.1. Materials

Gelatin from porcine skin Type A, carbonate–bicarbonate (CB) buffer, methacrylic anhydride (MAA) (94%), and dialysis cellulose membrane (12–14 kDa cutoff avg. flat width 25 mm) (94%) were supplied by Sigma Aldrich (St. Louis, MO, USA). Poly(L-lactide-co-ε-caprolactone) copolymer (PLCL, 70/30 LA/CL molar ratio) (PURASORB^®^ PLC 7015, inherent viscosity midpoint of 1.5 dL/g) was purchased from Corbion (Amsterdam, The Netherlands). Dichloromethane (DCM) and dimethylformamide (DMF) were supplied by Sigma Aldrich and used without further purification. Type I collagen solution from calf skin (acetic acid 0.25%) was obtained from Sigma Aldrich (St. Louis, MO, USA).

For the cell culture, human umbilical vein endothelial cells (HUVECs), Endothelial Cell Growth Medium with SupplementMix (EGM), and Detachkits were purchased from Promocell (Heidelberg, Germany). Biolaminin 411 for simulating the basal membrane of the intima tunica was supplied by Voden Medical Instruments (Meda, MB, Italy). The human oxidized low-density lipoprotein (ox-LDL) employed for inducing endothelial damage was supplied by Kalen Biomedical (Montgomery Village, MD, USA). CD31 (Dako, Glostrup, Denmark), F-actin and Alexa-Fluor 488 antibodies, Prolong Gold Antifade reagent with DAPI, and PrestoBlue™ cell viability reagent were obtained from ThermoFisher Scientific (Waltham, MA, USA). The Total Nitric Oxide and Nitrate/Nitrite Parameter Assay kit was purchased from Bio-Techne (Minneapolis, MN, USA). Hexamethyldisilazane was purchased from Merck (Darmstadt, Germany).

### 2.2. Synthesis of Gelatin Methacrylate (GelMa) and Hydrogel Preparation

GelMa was synthesized through a methacrylation reaction between the hydroxyl and primary amino groups of gelatin chains and MAA, following an optimized procedure [30,31]. The reaction was conducted under carefully controlled conditions, including alkaline pH, temperature, reagent concentration, and stirring. Specifically, a 10% (*w*/*v*) solution of gelatin type A (20 g) was prepared in 200 mL of 0.25 M CB buffer. The gelatin was dissolved with vigorous stirring for 1 h in a 500 mL two-neck round-bottom flask immersed in an oil bath and maintained at 50 °C. Methacrylation was initiated by adding MAA at a ratio of 0.1 mL per gram of gelatin while maintaining a temperature of 50 °C and stirring at 500 rpm. The reaction was carried out for 1 h, during which periodic additions of 5 M NaOH were made to maintain the pH at 9, as methacrylic acid, a byproduct of the functionalization, was released. The reaction was quenched by adding a few drops of 37% (*w*/*v*) HCl to adjust the pH to 7.4. Residual byproducts were removed by purifying the solution through dialysis with a 12–14 kDa molecular weight cutoff membrane for 5 days. The purified GelMa was subsequently lyophilized, yielding a white porous solid, which was stored at 4 °C for further use.

The GelMa hydrogel was prepared using a sterile 5 mL disposable syringe fitted with a female/female Luer lock adapter, which facilitated the connection to a second syringe for homogenization, resulting in a 5% (*w*/*v*) solution. After dissolution, the photoinitiator Irgacure 2959 was incorporated into the GelMa solution at a concentration of 0.2% (*w*/*v*). Photocrosslinking was achieved by exposing the solution to UV light (365 nm) with an intensity of 12 mW/cm^2^ for 120 s.

The successful synthesis of GelMa was confirmed through Fourier transform infrared spectroscopy (FT-IR) analyses. FT-IR was carried out using a Spectrum Two instrument equipped with an attenuated total reflectance-Fourier transform infrared (ATR) accessory (Perkin-Elmer, diamond crystal, Milan, Italy) on both the gelatin and GelMa. All spectra were registered between 800 cm^−1^ and 4000 cm^−1^, with a resolution of 4 cm^−1^, an accumulation of 16 scans, and a step size of 1 cm^−1^. A methacrylation degree (MD) was calculated by ^1^H-NMR spectrometry, following the method reported in the literature [32]. Analyses were conducted on a Bruker Advance III 400 spectrometer using 20 mg of GelMA dissolved in 0.70 mL of D_2_O. The new signals observed at δ = 5.4 and 5.7 ppm, corresponding to the acrylic protons of the methacrylic groups in the methacrylic anhydride structure, validated the synthesis. The methacrylation degree was calculated based on the decrease in intensity of the arginine methyl group signal at δ = 2.85 ppm using the following Equation (1):

(1) $$\mathrm{Methacrylation}\;\mathrm{degree}\;\left(\mathrm{mol}\%\right)\;\mathrm{or}\;MD\;=\;1\;-\;\frac{\mathrm{integration}\;\mathrm{signal}\;\mathrm{of}\;\mathrm{arginine}\;\mathrm{from}\;\mathrm{GelMa}\;}{\mathrm{integration}\;\mathrm{signal}\;\mathrm{of}\;\mathrm{argine}\;\mathrm{from}\;\mathrm{Gelatin}\;}\times \;100$$

### 2.3. Fabrication of Polymeric Nanofibrous Mats

A custom-built electrospinning apparatus was employed, consisting of a high-voltage power supply (Spellman SL 50 P 10/CE/230), a syringe pump (KD Scientific 200 series), and a glass syringe connected to a stainless steel blunt-ended needle. The polymer solution was electrospun onto a rotating metallic drum collector (length = 120 mm, diameter = 50 mm) designed to produce scaffolds composed of uniaxially aligned nanofibers. Electrospinning was performed under controlled conditions at room temperature (RT) and a relative humidity of 50%. Polymer solutions were prepared by dissolving PLCL in a solvent mixture of DCM and N,N-DMF at a 65:35 *v*/*v* ratio, with a polymer concentration of 20% *w*/*v*. The polymer solution was delivered at a constant flow rate (1.2 mL/h) through the needle, positioned 20 cm away from the collector. A voltage of 20 kV was applied to initiate fiber formation. Aligned nanofibers were produced by setting the collector to rotate at a linear speed of 6000 rpm. After 120 min of electrospinning, a mat with a thickness of 40–50 µm and dimensions of 15 × 8 cm was obtained. This procedure was optimized and described in detail in a previous study [13], which was used as a basis for this work.

### 2.4. Rheology of GelMa Hydrogels

Rheological analyses on uncrosslinked (u-GelMa) and crosslinked (c-GelMa) GelMa hydrogels were conducted using an MCR 102 rheometer (Anton Paar) in a parallel-plate configuration with a PP-25 geometry (plate diameter: 25 mm, gap: 0.3 mm). Once the sample was in contact with the upper plate, the excess material was carefully removed with a spatula, and the surrounding trap was filled with distilled water to mitigate evaporation during the measurement. Amplitude sweep tests were conducted to investigate the viscoelastic properties of both uncrosslinked and crosslinked samples, with the crosslinked samples incubated in phosphate-buffered saline (PBS) at 37 °C to simulate physiological conditions. Rheological measurements were performed after specific time intervals, including a few minutes post-immersion and after 24, 48, and 72 h of incubation. During the tests, the hydrogel was subjected to a strain range from 0.01% to 1000%, maintaining a constant angular frequency at 1 rad/s to evaluate the storage modulus (G′) and the loss modulus (G′′) as a function of the applied strain (%). Flow curve tests were conducted to evaluate the processability and manufacturability of the u-GelMa hydrogel. The tests were performed in the rotational mode by applying a shear rate ranging from 0.1 ^s−1^ to 1000 ^s−1^ to examine the material’s viscosity as a function of both shear rates.

### 2.5. Composite Sandwich Assembling

**Figure 1:**
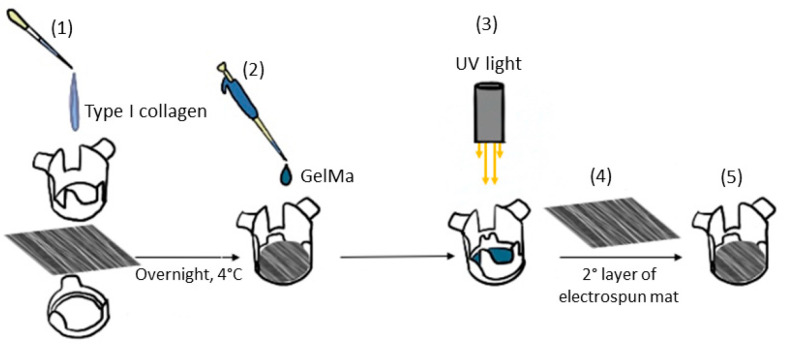
Schematic illustration of the procedure for the fabrication of a sandwich composite material. An aligned electrospun mat is placed at the bottom of a cell crown, followed by the addition of type I collagen (step 1) and GelMa (step 2). Photocrosslinking of GelMa using UV light (step 3). A second layer of electrospun mat is then added on top (step 4), resulting in a sandwich composite material within the cell crown (step 5).

Electrospun mats were prepared for cell seeding by cutting them into appropriately sized pieces and assembling them using CellCrown™ supports (Scaffdex, Tampere, Finland). The mats were sterilized with ethanol following a previously established protocol [33] and subsequently coated with 0.01% type I collagen solution prepared by diluting collagen from calf skin 10-fold with sterile water. The collagen coating was applied overnight at 4 °C, after which the solution was removed, and the scaffolds were washed with PBS. After sterilization and functionalization, 500 µL of u-GelMa hydrogel was dispensed onto the mat surface and photocrosslinked within the fibers, following a previously described protocol. A second electrospun mat was then layered on top of the GelMa-coated mat, forming a composite scaffold with a sandwich-like structure (Figure 1). Before the cell seeding, the upper mat of each sandwich-like structure was covered with biolaminin using a working concentration of 1 µg/cm^2^ for 2 h in an incubator at 37 °C and 5% CO_2_.

### 2.6. Dynamic Conditions with the LiveBox IVTech Bioreactor

The LiveBox bioreactor system (IVTech, Massarosa, Italy) was employed to facilitate dynamic, physiologically relevant conditions for the 3D culture of cells. The system features modular components, including a microfluidic system with hosting chambers and microtubing, allowing for the precise control of flow rates, shear stress, and media composition. This advanced device is specifically designed for interconnected dynamic cell cultures, incorporating a dual-chamber system (upper and lower chambers) with flow inlets and outlets and a holder for a porous PET membrane; then, the composite scaffolds were placed inside the hosting chambers. The bioreactor was connected to a programmable peristaltic pump, ensuring stable laminar flow at 50 µL/min and 500 µL/min flow rates, and maintained at 37 °C in a humidified environment with 5% CO_2_ for 24 h.

### 2.7. Cell Culture and Treatments

**Figure 2:**
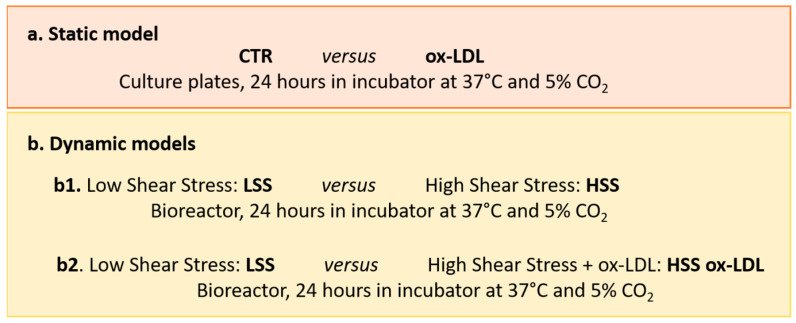
Experimental setup of the study. (**a**) HUVECs cultured on sandwich-like scaffolds in static and (**b**) dynamic conditions. CTR and LSS: cells cultured in EGM culture medium; ox-LDL and HSS ox-LDL: cells exposed to oxidized low-density lipoprotein added to EGM. LSS: low shear stress (50 µL/min flow rate); HSS: high shear stress (500 µL/min flow rate).

HUVECs were cultured in complete EGM and expanded until confluence, changing the medium every 2–3 days. For the static experiments, 2 × 10^5^ cells were seeded on each sandwich-like scaffold and incubated at 37 °C and 5% CO_2_ overnight to allow for cell adhesion. The following day, we induced the endothelial damage by treating HUVECs with 100 µg/mL of human ox-LDL added to complete EGM for 24 h (ox-LDL); the control cells were cultured in EGM without ox-LDL (CTR). For the dynamic experiments, 3.5 × 10^5^ cells were seeded on scaffolds and left to adhere overnight in an incubator. After 24 h, scaffolds were placed in the bioreactor chambers according to the following experimental scheme: low shear stress (LSS) at a 50 µL/min flow rate and high shear stress (HSS) at a 500 µL/min flow rate; HSS with/without ox-LDL (Figure 2). At the end of each experimental condition, the samples were processed for cellular and ultrastructural analysis, while the supernatants were collected and stored at −80 °C for an ELISA analysis.

### 2.8. Cell Viability

The HUVEC viability under culture on each sandwich-like scaffold in static and dynamic conditions was assessed using a commercial Presto Blue assay, in accordance with the manufacturer’s datasheet. Briefly, the medium of each scaffold was exchanged with a solution composed of fresh EGM with 10% of Presto Blue and incubated at 37 °C for 4 h. Then, 100 μL taken from each CTR and ox-LDL sandwich-like scaffold cultured in static and dynamic conditions was dispensed in a 96-well plate in triplicate, measuring the absorbance at 490 nm through a Multiskan SkyHigh (ThermoFisher Scientific) microplate reader.

### 2.9. Immunofluorescence

The endothelial and cytoskeleton molecule expression was evaluated using an immunofluorescence assay. Scaffolds cultured in static conditions were fixed with a solution of 2% paraformaldehyde in PBS with or without 1% Triton X-100 for 4 min at RT. Then, the samples were blocked with a solution of 1% bovine serum albumin (BSA) in PBS for 30 min at RT and subsequently labeled with CD31 (1:80, Dako, Glostrup, Denmark) primary antibody for 1 h, incubated at 37 °C, and Alexa Fluor 488 Phalloidin (1:500; ThermoFisher Scientific) fluorescent conjugated antibody for 30 min at RT. After several washings with PBS, scaffolds stained with CD31 were labeled with the Alexa Fluor 488 (1:250, ThermoFisher Scientific) secondary antibody for 1 h at 37 °C; all incubations were performed in a wet chamber in the dark, and all antibodies were diluted in 1% PBS/BSA. Then, the samples were rinsed and counterstained with Prolong Gold Antifade reagent with DAPI before the observation. The scaffolds were examined in a spectral confocal microscope (Nikon A1R mod. Ti2 Eclipse), acquiring digital images.

### 2.10. ELISA Assay to Determine the NO Levels

Nitric oxide (NO) levels were determined on culture media collected from HUVEC sandwich-like scaffolds in static and dynamic conditions, as summarized in Figure 2. Cell culture supernatants were centrifuged at 1500 rpm for 10 min to remove particulates and debris.

The NO concentration was determined using a Total Nitric Oxide and Nitrate/Nitrite Assay, according to the manufacturer’s instructions. The optical density (OD) of each sample was determined using a Multiskan SkyHigh (ThermoFisher Scientific) microplate reader set at 540 nm with a wavelength correction at 690 nm. A standard curve was obtained from serial dilutions of NO standards, according to the manufacturer’s instructions.

### 2.11. Scanning Electron Microscopy

A scanning electron microscopy (SEM) analysis was carried out by using a desktop SEM (INCAx sight, Oxford Instrument, model: 7060; Abingdon, UK) set at a voltage of 20 kV on the samples fixed with a conducting bi-adhesive tape on an aluminum stub and coated with gold (by S150A Sputter Coater, Edwards, Burgess Hill, UK). The images were analyzed with the Image J software (version 1.53k, National Institutes of Health, USA), and the porosity was determined by measuring about 300 pores. The results are presented as the average pore sizes ± standard deviation.

SEM was also used to examine the HUVEC cells cultured on sandwich-like scaffolds in static and dynamic conditions with/without exposure to ox-LDL. After washing with PBS, the samples were fixed in 2.5% buffered glutaraldehyde overnight at 4 °C and subsequently post-fixed in 1% osmium tetroxide for 15 min. Then, the samples were washed in distilled water and dehydrated with ethanol at increasing concentrations (70%, 95%, and 100%) for 15 min for each passage. For the dry specimens, they were immersed in a 1:1 solution of absolute ethanol and hexamethyldisilazane (HMDS) and then in pure HMDS for 30 min each and finally air-dried; after glutaraldehyde fixation, all steps were performed at RT. Before the SEM analysis, the scaffolds were attached to aluminum stubs (Multilab type stub pin ^1^/_2_, Surrey, UK) using a conducting bi-adhesive tape for electron microscopy, coated with gold with the S150A Sputter Coater, and observed at 10 kV in an SEM microscope (Thermo Scientific Quattro S, Waltham, MA, USA) with secondary electrons.

### 2.12. Statistical Analysis

The GraphPad Prism 8 software was used for the statistical analysis and graph creations. Statistical differences between the control (CTR and LSS) and treated samples (ox-LDL, HSS, and HSS ox-LDL) were evaluated using an unpaired Student’s *t*-test. Each experiment was performed in duplicate or triplicate. The values are expressed as means ± standard deviation; a *p*-value < 0.05 was considered statistically significant.

## 3. Results

### 3.1. Polymer, GelMa, and Scaffolds Characterization

Polymeric scaffolds composed of the PLCL 70/30 copolymer were fabricated via electrospinning, resulting in nanofibrous porous mats using an optimized procedure described in the Materials and Methods section [13]. The aim was to reproduce the structure and mechanical properties of the internal elastic lamina of the tunica intima. This procedure ensured bead-free fibers, with an average diameter of 0.7 ± 0.2 µm. The SEM analysis confirmed uniform and well-aligned fibers, with 68% of the nanofibers oriented within a range of 0–18° from the collector’s rotation direction. The fibers’ morphology remained stable after sterilization and a thermal treatment. Coating the scaffold with type I collagen significantly improved its hydrophilicity, reducing the water contact angle (WCA) from 98.72° to 37.9° [13].

GelMa was utilized to replicate the native human sub-endothelium of the tunica intima. FT-IR spectroscopy was conducted to analyze both pristine gelatin and gelatin methacrylate (GelMa), confirming the successful substitution of functional groups during methacrylation. The spectra (Figure S1a) revealed characteristic peaks of the gelatin polymer, including a broad absorption band at 3340 cm^−1^ attributed to O-H and N-H stretching vibrations and peaks in the region of 2800–3100 cm^−1^ assigned to C-H stretching vibrations. Backbone-related absorption bands were observed at 1630 cm^−1^ (C=O stretching, amide I), 1540 cm^−1^ (N-H bending coupled with C-H stretching, amide II), and 1250 cm^−1^ (C-N stretching and N-H bending, amide III). Upon methacrylation, the spectrum of GelMa reveals distinct changes. A new peak emerges at approximately 1720 cm^−1^, corresponding to the stretching vibration of the ester carbonyl (C=O) group introduced by the methacrylate groups. Moreover, a methacrylation degree of 75% was calculated by ^1^H-NMR (Figure S1b) using Equation (1).

The morphological characteristics of u-GelMa and c-GelMa hydrogels were evaluated using SEM to assess the impact of the crosslinking on their structural properties. As shown in Figure S1c, u-GelMa exhibited a relatively smooth surface morphology, reflective of a loosely organized internal structure with limited porosity. This compact morphology suggests a lower capacity for cellular infiltration or nutrient transport. In contrast, c-GelMa displayed a highly porous structure with a well-defined network of interconnected pores. A quantitative analysis of the pore structure revealed an average pore size of 12.27 ± 0.35 µm, with a uniform size distribution. The presence of these pores is a critical feature for enabling effective nutrient and oxygen diffusion. The significant morphological transformation observed following crosslinking can be attributed to the formation of covalent bonds between acrylate moieties in the GelMA structure, which stabilize the hydrogel network while introducing controlled porosity.

**Figure 3:**
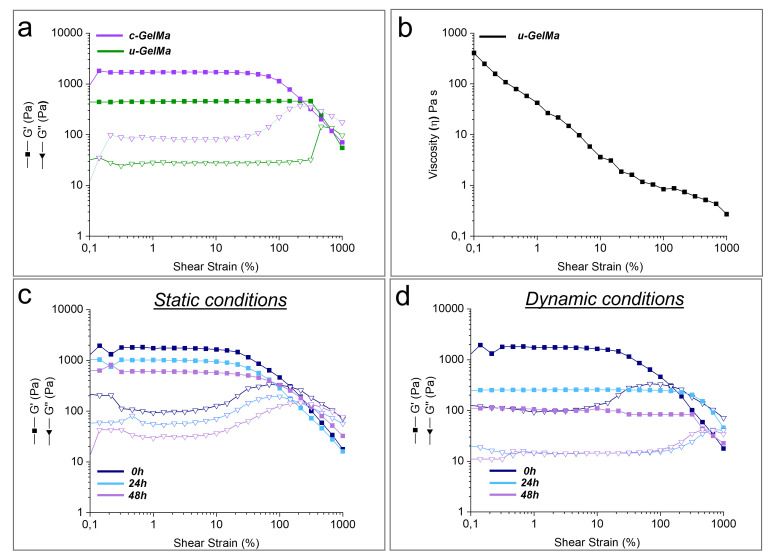
Rheological characterization of uncrosslinked (u-GelMa) and crosslinked GelMa (c-GelMa). (**a**) Amplitude sweep test of u-GelMa and c-GelMa at 25 °C, showing storage modulus (G′) and loss modulus (G′) as a function of the applied shear strain. (**b**) Viscosity curve of u-GelMa, illustrating viscosity as a function of the applied shear rate. Amplitude sweep tests of c-GelMa at 37 °C at different time points (0 h, 24 h, and 48 h) under (**c**) static and (**d**) dynamic conditions, respectively.

To evaluate the rheological properties of GelMa hydrogels, oscillatory and rotational rheological analyses were conducted. These tests assessed the storage modulus (G′), loss modulus (G″), and viscosity, all critical parameters for reproducing the mechanical and viscoelastic environments of the extracellular matrix of sub-endothelial tissue. Oscillatory amplitude sweep tests revealed how the G′ and G″ varied after crosslinking as a function of strain (0.1–1000%). Figure 3a compares u-GelMa and c-GelMa at 25 °C, showing a significant increase in G′, which reaches approximately 1703 Pa after crosslinking, indicating enhanced stiffness of the hydrogel.

Rotational rheology was employed to evaluate the viscosity profile of GelMa as a function of the shear rate, providing insights into its injectability. As shown in Figure 3b, the viscosity decreases from 405 Pa s at 0.1 s^−1^ to 0.2 Pa s at 1000 s^−1^. This behavior confirmed that GelMA exhibits shear-thinning properties, a crucial characteristic for its injectability and its ability to form thin, precise hydrogel layers.

Additionally, c-GelMa hydrogels were subjected to stability tests under static and dynamic conditions to assess their rheological properties in a physiological environmental condition (T = 37 °C). Amplitude sweep measurements were performed after 24 and 48 h to evaluate the hydrogel’s mechanical behavior over time. These time points were selected due to the necessity to preserve the integrity of scaffolds to conduct biological assays. Figure 3c reveals that c-GelMa, under static conditions, retained its gel-like structure for up to 48 h, with a slight decrease in G′ and G″ values over time (Table S2). Figure 3d presents the results of the same amplitude sweep analysis conducted under dynamic conditions. The dynamic environment caused a more pronounced decrease in mechanical properties compared to static conditions, as evidenced by the larger reduction in G′ and G″ values (Table S2). Despite this decline, c-GelMa maintained its gel-like behavior, demonstrating its ability to withstand the mechanical challenges posed by dynamic conditions while preserving its structural integrity.

### 3.2. Biological Evaluation of the Sandwich-like Scaffolds with HUVECs in Static Conditions

The assembly of the composite material followed a stepwise process, as described in the Materials and Methods section. A nanofibrous PLCL mat coated with type I collagen was layered with c-GelMa, which was subsequently covered with a second nanofibrous PLCL mat, forming a sandwich-like structure.

**Figure 4:**
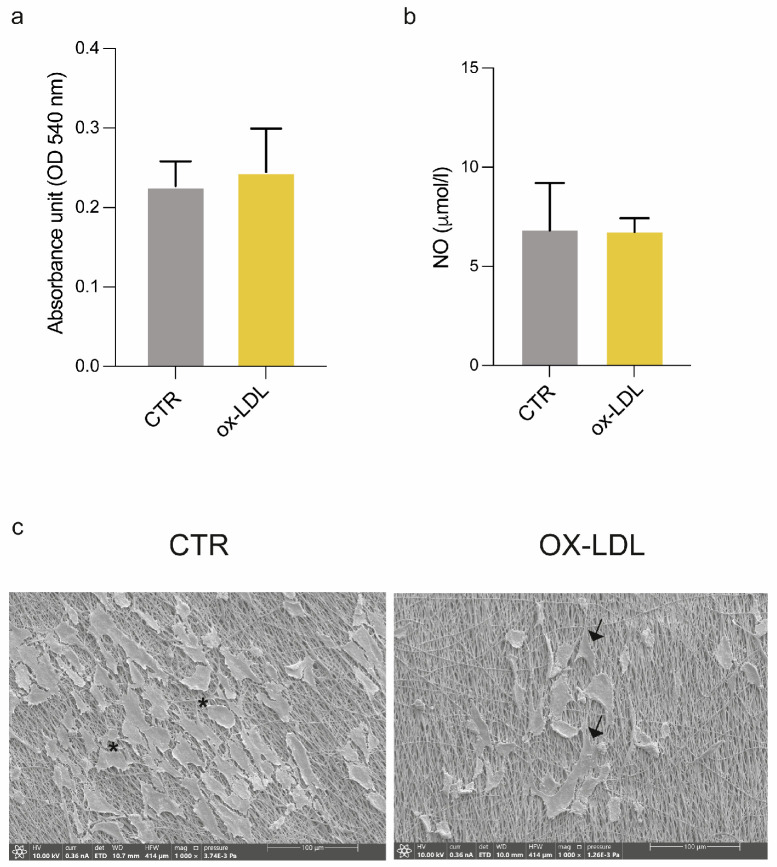
Effects of ox-LDL administration in HUVECs in the 3D model under static conditions. (**a**) HUVEC viability on the 3D composite model. Analysis of (**b**) NO protein levels in HUVEC supernatants by ELISA assays. (**c**) Representative ultrastructural images of HUVECs (CTR and ox-LDL) cultured on sandwich-like scaffolds. Spindle-shaped cells: asterisks; rounded cells: arrows. Scale bar images: 100 μm. *, *p* < 0.05, unpaired Student’s *t*-test. ox-LDL: oxidized low-density lipoprotein; CTR: cells cultured in EGM; ox-LDL: cells treated with ox-LDL added to culture medium; NO: nitric oxide. Scale bars images: 100 μm.

In static experiments, the cell viability analysis of HUVECs seeded on this 3D model of vascular intimal tunica performed with a Presto Blue assay does not show significant variations between the CTR and ox-LDL samples, despite a slight increase observed in samples exposed to ox-LDL (Figure 4a). The analysis of cell culture supernatants did not reveal significant changes in NO protein levels following the treatment (Figure 4b). Ultrastructural analysis performed with SEM on the 3D model generated showed HUVEC adhesion to the nanostructured sandwich-like scaffolds in both CTR and ox-LDL experimental conditions (Figure 4c). Moreover, some alterations in HUVEC cell morphology were observed; in particular, some cells displayed a spindle morphology with the presence of cytoplasmic prolongations in contrast to the typical cobblestone morphology of endothelial cells. A confocal microscopy analysis revealed a reduction in CD31 expression in HUVECs cultured on the 3D model under the ox-LDL treatment (Figure S2). Furthermore, an analysis of F-actin was performed to investigate the cytoskeletal architecture. As shown in Figure S2a, HUVECs treated with ox-LDL underwent a cytoskeletal remodeling of actin filaments, functional to the change in morphology toward a spindle-shaped and fibroblast-like trend (Figure S2b).

**Figure 5:**
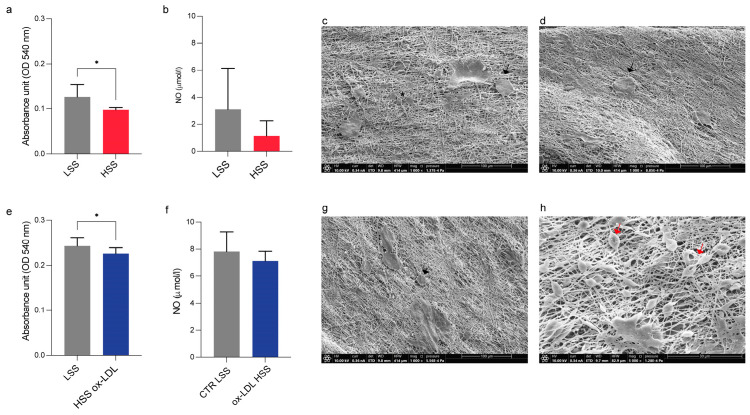
Cell viability, morphology, and released proteins of HUVECs in the 3D model in dynamic conditions. (**a**) Viability; (**b**) NO levels; and (**c**,**d**) morphology of HUVECs cultured in the 3D composite model in a bioreactor system after exposure to LSS and HSS; (**e**) viability; (**f**) NO levels; and (**g**,**h**) morphology of HUVECs cultured in the 3D composite model in a bioreactor system after exposure to HSS and treated with ox-LDL. For (**c**,**d**,**g**,**h**): spindle-shaped (asterisks), rounded (arrows), and red blood cell-like (red arrows) morphologies. Scale bar images: 100 μm for (**c**,**d**,**g**); 30 μm for (**h**). ox-LDL: oxidized low-density lipoprotein; LSS: low shear stress; HSS: high shear stress; HSS ox-LDL: HUVECs treated with ox-LDL added to culture medium. * *p*-values < 0.05 (0.0271 in (**a**) and 0.041 in (**e**)), unpaired Student’s *t*-test.

Under dynamic conditions, the cell viability of HUVECs cultured in a bioreactor system and exposed to different flow rates showed a significant reduction in the HSS condition compared to those in the LSS condition (Figure 5a). Instead, when cultured in the same experimental conditions with the addition of ox-LDL in the HSS, their viability was slightly lower than their LSS counterpart (Figure 5e). The measurement of NO in the cell culture medium did not show significant differences, other than a decreasing trend in HUVECs exposed to HSS in both experimental settings (Figure 5b,f). Ultrastructural examination performed on HUVECs seeded on the 3D nanostructured composite model revealed the attachment of HUVECs to sandwich-like scaffolds in all dynamic culture conditions (LSS, HSS, and HSS ox-LDL), with changes in morphology, such as elongated and rounded cells with long cytoplasmic projections (Figure 5c,d); for the HSS ox-LDL condition, HUVECs displayed a rounded shape (Figure 5g) and, interestingly, also a red blood cell-like morphology (Figure 5h).

## 4. Discussion

The PLCL 70/30 scaffolds exhibited physicochemical and mechanical properties suitable for applications aimed at mimicking the internal elastic membrane of tunica intima [9]. The high degree of nanofiber alignment (68% within 0–18°), achieved during the electrospinning process, is particularly significant for recreating the anisotropic structure of the internal elastic membrane [34], which plays a crucial role in tissue functionality. Furthermore, the stability of the fiber morphology after sterilization and a thermal treatment ensures the reliability of the scaffold under experimental and physiological conditions. The addition of type I collagen not only improved the biocompatibility but also significantly enhanced the hydrophilicity, as evidenced by the reduction in WCA, thus favoring cellular adhesion and proliferation [13]. The coating with collagen type I was also critical for promoting stable integration with the GelMa hydrogel. The presence of collagen introduces reactive hydrophilic functional groups, which enhance physical interactions with GelMa, improving the adhesion and composite integrity. Beyond their structural advantages, PLCL nanofibers have also been shown to support co-culture strategies for vascular tissue engineering. For instance, Lee et al. [35] demonstrated that electrospun PLCL scaffolds can be used to seed endothelial and smooth muscle cells, effectively recapitulating the layered configuration of native blood vessels. This provides a promising basis for future adaptations involving multiple vascular cell types, further increasing the physiological relevance of our engineered construct.

The FT-IR analysis confirmed that GelMa retains key structural features of pristine gelatin while introducing methacrylate groups. The appearance of the 1720 cm^−1^ peak in the GelMa spectrum, absent in the native gelatin, is particularly significant, as it signifies the introduction of ester bonds from methacrylate groups. Complementary to the FT-IR findings, the degree of methacrylation was quantified using ^1^H-NMR, revealing a methacrylation efficiency.

The morphological characteristics of u-GelMa and c-GelMa hydrogels were examined through SEM to evaluate how crosslinking influences their structural properties. U-GelMa displayed a relatively smooth surface morphology, indicative of a loosely structured internal network with minimal porosity. This compact morphology limits its ability to support cellular infiltration or efficient nutrient transport. On the other hand, c-GelMa exhibited a highly porous structure, characterized by a clear network of interconnected pores. This porosity is essential for promoting the effective diffusion of nutrients and oxygen, as well as supporting cell migration and infiltration within the hydrogel [36]. The notable morphological shift observed post-crosslinking is attributed to the formation of covalent bonds between the acrylate groups in the GelMA, which not only stabilize the hydrogel network but also introduce controlled porosity to enhance its functional properties.

The rheological analyses of GelMA hydrogels demonstrated their ability to replicate key mechanical and viscoelastic properties of the ECM of the sub-endothelial layer in the tunica intima. The significant increase in the storage modulus after crosslinking highlights the enhanced stiffness and mechanical stability of the hydrogel. This increase reflects the formation of a robust crosslinked network, which is critical for maintaining structural integrity under physiological conditions (Table S1). These values indicate that c-GelMa initially presents a stiffness slightly higher than that of the native vascular basement membrane (BM), whose reported elastic modulus ranges from 0.1 kPa to 1 kPa [37]. However, rheological properties under culture conditions revealed a progressive decrease in the elastic modulus (G′) over time, particularly under dynamic flow (Table S2), thereby approaching the mechanical range of the native BM. This time-dependent softening is particularly relevant, as it allows the hydrogel to transition toward a more compliant, biomimetic environment, which promotes proper cell functions.

Additionally, the observed shear-thinning behavior is another essential feature of GelMa hydrogels, as it facilitates their injectability and precise deposition. The decrease in viscosity from 405 Pa·s at 0.1 s^−1^ to 0.2 Pa·s at 1000 s^−1^ underscores the material’s ability to flow under applied shear stress, allowing it to form thin, uniform layers during deposition through a nozzle. The rheological stability of c-GelMa under both static and dynamic conditions highlights its potential for replicating physiological environments. The retention of gel-like behavior at 37 °C for up to 48 h under static conditions suggests that c-GelMa hydrogels possess sufficient mechanical integrity to support cellular and tissue interactions over short-term experimental time frames. The slight reduction in G′ and G″ values observed over time reflects a minor relaxation of the crosslinked network, which is expected as the hydrogel equilibrates with the surrounding environment. Under dynamic conditions, however, the hydrogel experienced a more pronounced decrease in mechanical properties, likely due to increased shear forces and mechanical stresses encountered during bioreactor operations. This reduction in G′ and G″ values underscores the impact of dynamic environments on the structural stability of hydrogels. Nevertheless, the hydrogel’s ability to preserve its gel-like characteristics, even under these more challenging conditions, demonstrates its capability to preserve the structural integrity of the scaffold.

To gain deep knowledge on the endothelial dysfunction and the early stage of atherosclerosis development, several 3D in vitro models have been developed [38,39] to overcome the limitations of 2D in vitro cellular systems, considered too simplified and unable to reproduce the cell−matrix interactions. In this contest, the sandwich-like configuration of our composite model was designed to mimic the multilayered organization of the human tunica intima, combining the mechanical strength and anisotropy of the PLCL nanofibrous mats with the viscoelastic properties and porosity of the c-GelMa hydrogel. According to our data, the 3D supports were suitable for cell culture and did not affect HUVEC viability and NO secretion, even in the presence of ox-LDL, a well-known trigger of endothelial damage and atherosclerosis initiation. The best experimental setting able to reproduce the endothelial dysfunction was the dynamic system induced by HUVECs grown under HSS in combination with ox-LDL. Shear stress is a well-known contributing factor to atherosclerosis, affecting endothelial cell gene expression and mechanical properties [40]. Indeed, we found a detrimental effect of HSS on cell viability, both in standard growth medium and with ox-LDL addition. Unfortunately, NO data were not significant and only highlighted a decreasing trend following HSS and HSS culture with ox-LDL. According to the literature, ox-LDL and NO exert opposite effects on the human endothelium: ox-LDL promotes endothelial damage, atherosclerosis initiation, and all of the related downstream processes; conversely, NO performs a protective and anti-atherogenic role on the vasculature [41].

## 5. Conclusions

The present study supports the suitability of a 3D composite scaffold for cell culture to mimic the multilayer structure of the human tunica intima without compromising HUVEC cell viability. The application of this 3D model for studying endothelial dysfunction and atherosclerosis initiation requires further investigations for establishing the appropriate experimental setting. Based on our results, dynamic culture with HSS represents the highest trigger of HUVEC alterations in terms of cell viability and morphology, especially under an ox-LDL treatment. The improvement and optimization of the experimental setup, also including multiple cell types for cell–cell interactions, are the necessary pillars of future investigations for validating the 3D model in atherosclerosis research.

## Data Availability

All relevant data are available within the manuscript.

## Supplementary Materials

The following are available online at https://www.mdpi.com/article/10.3390/biom15060865/s1, Figure S1: **a)** FTIR spectra of gelatine (orange) and GelMa (red) in the range of 4000–800 cm^−1^; **b)** Representative ^1^H-NMR spectra of gelatine and GelMa. **c)** SEM images of hydrogel samples: uncrosslinked (u-GelMa) and crosslinked GelMa (c-GelMa), Figure S2: CD31 expression and remodeling of F-actin filaments of HUVEC in the 3D model of vascular intima tunica, Table S1: Summary of the elastic (G′) and loss (G″) moduli, crossover point for hydrogel samples at 25 °C (*Amplitude Sweep Test*), Table S2: Summary of the elastic (G′) and loss (G″) moduli, crossover point for c-GelMa at 37 °C (*Amplitude Sweep Test*).

## Abbreviations

CB	carbonate-bicarbonate
c-GelMa	crosslinked GelMa
CTR	control cells
DCM	dichloromethane
DMF	dimethylformamide
ECs	endothelial cells
ECM	extracellular matrix
EGM	endothelial growth medium
ELISA	enzyme-linked immunosorbent assay
FT-IR	Fourier transform infrared spectroscopy
GelMa	methacrylated gelatin hydrogel
HCL	hydrochloric acid
HMDS	hexamethyldisilazane
HSS	high shear stress
HUVECs	human umbilical vein endothelial cells
LDL	low-density lipoprotein
LSS	low shear stress
MAA	methacrylic anhydride
MD	methacrylation degree
NO	nitric oxide
OD	optical density
ox-LDL	oxidized low-density lipoprotein
PBS	phosphate-buffered saline
PLCL	poly(L-lactide-co-ε-caprolactone) copolymer
RT	room temperature
SEM	scanning electron microscopy
SMCs	smooth muscle cells
u-GelMa	uncrosslinked GelMa
UV	ultraviolet light
WCA	water contact angle

## References

[1] Davignon J., Ganz P. (2004). Role of Endothelial Dysfunction in Atherosclerosis. Circulation.

[2] Higashi Y. (2022). Roles of Oxidative Stress and Inflammation in Vascular Endothelial Dysfunction-Related Disease. Antioxidants.

[3] Andrabi S.M., Sharma N.S., Karan A., Shahriar S.M.S., Cordon B., Ma B., Xie J. (2023). Nitric Oxide: Physiological Functions, Delivery, and Biomedical Applications. Adv. Sci..

[4] Denicola A., Batthyány C., Lissi E., Freeman B.A., Rubbo H., Radi R. (2002). Diffusion of Nitric Oxide into Low Density Lipoprotein*. J. Biol. Chem..

[5] Pepin M.E., Gupta R.M. (2024). The Role of Endothelial Cells in Atherosclerosis: Insights from Genetic Association Studies. Am. J. Pathol..

[6] Kardassis D., Vindis C., Stancu C.S., Toma L., Gafencu A.V., Georgescu A., Alexandru-Moise N., Molica F., Kwak B.R., Burlacu A. (2025). Unravelling Molecular Mechanisms in Atherosclerosis Using Cellular Models and Omics Technologies. Vasc. Pharmacol..

[7] Foglietta F., Canaparo R., Muccioli G., Terreno E., Serpe L. (2020). Methodological Aspects and Pharmacological Applications of Three-Dimensional Cancer Cell Cultures and Organoids. Life Sci..

[8] Chan B.P., Leong K.W. (2008). Scaffolding in Tissue Engineering: General Approaches and Tissue-Specific Considerations. Eur. Spine J..

[9] Youn J., Han H., Park S.M., Kim D.S. (2021). Arterial Internal Elastic Lamina-Inspired Membrane for Providing Biochemical and Structural Cues in Developing Artery-on-a-Chip. ACS Macro Lett..

[10] Mu X., Gerhard-Herman M.D., Zhang Y.S. (2023). Building Blood Vessel Chips with Enhanced Physiological Relevance. Adv. Mater. Technol..

[11] Pagnotta G., Kalia S., Di Lisa L., Cicero A.F.G., Borghi C., Focarete M.L. (2022). Progress towards 3D Bioprinting of Tissue Models for Advanced Drug Screening: In Vitro Evaluation of Drug Toxicity and Drug Metabolism. Bioprinting.

[12] Gualandi C., Govoni M., Foroni L., Valente S., Bianchi M., Giordano E., Pasquinelli G., Biscarini F., Focarete M.L. (2012). Ethanol Disinfection Affects Physical Properties and Cell Response of Electrospun Poly(l-Lactic Acid) Scaffolds. Eur. Polym. J..

[13] Pacilio S., Costa R., Papa V., Rodia M.T., Gotti C., Pagnotta G., Cenacchi G., Focarete M.L. (2023). Electrospun Poly(L-Lactide-Co-ε-Caprolactone) Scaffold Potentiates C2C12 Myoblast Bioactivity and Acts as a Stimulus for Cell Commitment in Skeletal Muscle Myogenesis. Bioengineering.

[14] Gogoi D., Kumar M., Singh J. (2024). A Comprehensive Review on Hydrogel-Based Bio-Ink Development for Tissue Engineering Scaffolds Using 3D Printing. Ann. 3D Print. Med..

[15] Im G.-B., Lin R.-Z. (2022). Bioengineering for Vascularization: Trends and Directions of Photocrosslinkable Gelatin Methacrylate Hydrogels. Front. Bioeng. Biotechnol..

[16] Kurian A.G., Singh R.K., Patel K.D., Lee J.H., Kim H.W. (2021). Multifunctional GelMA Platforms with Nanomaterials for Advanced Tissue Therapeutics. Bioact. Mater..

[17] Chen Y.C., Lin R.Z., Qi H., Yang Y., Bae H., Melero-Martin J.M., Khademhosseini A. (2012). Functional Human Vascular Network Generated in Photocrosslinkable Gelatin Methacrylate Hydrogels. Adv. Funct. Mater..

[18] Wang Y., Huang N., Yang Z. (2023). Revealing the Role of Zinc Ions in Atherosclerosis Therapy via an Engineered Three-Dimensional Pathological Model. Adv. Sci..

[19] Pham Q.P., Sharma U., Mikos A.G. (2006). Electrospun Poly(Epsilon-Caprolactone) Microfiber and Multilayer Nanofiber/Microfiber Scaffolds: Characterization of Scaffolds and Measurement of Cellular Infiltration. Biomacromolecules.

[20] Mi C.-H., Qi X.-Y., Zhou Y.-W., Ding Y.-W., Wei D.-X., Wang Y. (2024). Advances in Medical Polyesters for Vascular Tissue Engineering. Discov. Nano.

[21] Zhao L., Li X., Yang L., Sun L., Mu S., Zong H., Li Q., Wang F., Song S., Yang C. (2021). Evaluation of Remodeling and Regeneration of Electrospun PCL/Fibrin Vascular Grafts in Vivo. Mater. Sci. Eng. C Mater. Biol. Appl..

[22] Fu W., Liu Z., Feng B., Hu R., He X., Wang H., Yin M., Huang H., Zhang H., Wang W. (2014). Electrospun Gelatin/PCL and Collagen/PLCL Scaffolds for Vascular Tissue Engineering. IJN.

[23] Shafiq M., Jung Y., Kim S.H. (2015). Stem Cell Recruitment, Angiogenesis, and Tissue Regeneration in Substance P-Conjugated Poly(l-Lactide-Co-ɛ-Caprolactone) Nonwoven Meshes. J. Biomed. Mater. Res. Part A.

[24] Kim T.H., Yan J.-J., Jang J.Y., Lee G.-M., Lee S.-K., Kim B.S., Chung J.J., Kim S.H., Jung Y., Yang J. (2021). Tissue-Engineered Vascular Microphysiological Platform to Study Immune Modula-tion of Xenograft Rejection. Sci. Adv..

[25] Kim D., Chung J.J., Jung Y., Kim S.H. (2019). The effect of Substance P/Heparin conjugated PLCL polymer coating of bioinert ePTFE vascular grafts on the recruitment of both ECs and SMCs for accelerated regeneration. Sci. Rep..

[26] Wu T., Zhang J., Wang Y., Sun B., Guo X., Morsi Y., El-Hamshary H., El-Newehy M., Mo X. (2017). Development of Dynamic Liquid and Conjugated Electrospun Poly(L-Lactide-Co-Caprolactone)/Collagen Nanoyarns for Regulating Vascular Smooth Muscle Cells Growth. J. Biomed. Nanotechnol..

[27] Jiang H., Zhou Y., Nabavi S.M., Sahebkar A., Little P.J., Xu S., Weng J., Ge J. (2022). Mechanisms of Oxidized LDL-Mediated Endothelial Dysfunction and Its Consequences for the Development of Atherosclerosis. Front. Cardiovasc. Med..

[28] Zhou M., Yu Y., Chen R., Liu X., Hu Y., Ma Z., Gao L., Jian W., Wang L. (2023). Wall Shear Stress and Its Role in Atherosclerosis. Front. Cardiovasc. Med..

[29] Meng F., Cheng H., Qian J., Dai X., Huang Y., Fan Y. (2022). In Vitro Fluidic Systems: Applying Shear Stress on Endothelial Cells. Med. Nov. Technol. Devices.

[30] Shirahama H., Lee B.H., Tan L.P., Cho N.-J. (2016). Precise Tuning of Facile One-Pot Gelatin Methacryloyl (GelMA) Synthesis. Sci. Rep..

[31] Di Lisa L., Rea M., Nuvoli D., Focarete M.L., Albonetti C., Mariani A. (2024). Frontal Polymerization of Acrylamide/GelMA/Gelatin Hydrogels with Controlled Mechanical Properties and Inherent Self-Recovery. Eur. Polym. J..

[32] Leu Alexa R., Cucuruz A., Ghițulică C.-D., Voicu G., Stamat (Balahura) L.-R., Dinescu S., Vlasceanu G.M., Stavarache C., Ianchis R., Iovu H. (2022). 3D Printable Composite Biomaterials Based on GelMA and Hydroxyapatite Powders Doped with Cerium Ions for Bone Tissue Regeneration. Int. J. Mol. Sci..

[33] Alessandri M., Lizzo G., Gualandi C., Mangano C., Giuliani A., Focarete M.L., Calzà L. (2014). Influence of Biological Matrix and Artificial Electrospun Scaffolds on Proliferation, Differentiation and Trophic Factor Synthesis of Rat Embryonic Stem Cells. Matrix Biol..

[34] Mishani S., Belhoul-Fakir H., Lagat C., Jansen S., Evans B., Lawrence-Brown M. (2021). Stress Distribution in the Walls of Major Arteries: Implications for Atherogenesis. Quant. Imaging Med. Surg..

[35] Lee B., Shafiq M., Jung Y., Park J.-C., Kim S.H. (2016). Characterization and Preparation of Bio-Tubular Scaffolds for Fabricating Artificial Vascular Grafts by Combining Electrospinning and a Co-Culture System. Macromol. Res..

[36] Hu Z.-Y., Chen G., Yi S.-H., Wang Y., Liu Q., Wang R. (2021). Multifunctional Porous Hydrogel with Nutrient Controlled-Release and Excellent Biodegradation. J. Environ. Chem. Eng..

[37] Salimbeigi G., Vrana N.E., Ghaemmaghami A.M., Huri P.Y., McGuinness G.B. (2022). Basement Membrane Properties and Their Recapitulation in Organ-on-Chip Applications. Mater. Today Bio.

[38] Maringanti R., van Dijk C.G.M., Meijer E.M., Brandt M.M., Li M., Tiggeloven V.P.C., Krebber M.M., Chrifi I., Duncker D.J., Verhaar M.C. (2024). Atherosclerosis on a Chip: A 3-Dimensional Microfluidic Model of Early Arterial Events in Human Plaques. Arterioscler. Thromb. Vasc. Biol..

[39] Kong D., Ryu J.-C., Shin N., Lee S.-E., Kim N.G., Kim H.-Y., Kim M.-J., Choi J., Kim D.-H., Kang K.-S. (2025). In Vitro Modeling of Atherosclerosis Using iPSC-Derived Blood Vessel Organoids. Adv. Healthc. Mater..

[40] Zhou J., Li Y.-S., Chien S. (2014). Shear Stress-Initiated Signaling and Its Regulation of Endothelial Function. Arterioscler. Thromb. Vasc. Biol..

[41] Gradinaru D., Borsa C., Ionescu C., Prada G.I. (2015). Oxidized LDL and NO Synthesis—Biomarkers of Endothelial Dysfunction and Ageing. Mech. Ageing Dev..

