# Supplementary material for: A 3D Composite Model Using Electrospinning Technology to Study Endothelial Damage
Source: Biomolecules. 2025 Jun 13;15(6):865. doi: 10.3390/biom15060865 (PMC12190949; doi:10.3390/biom15060865)
Supplement: Supplementary file 1 [file biomolecules-15-00865-s001.zip › Supplementary materials_Ciavarella C et al.pdf]

biomolecules

MDPI

Article

A 3D composite model using electrospinning technology to study the endothelial damage

Carmen Ciavarella [1,‡], Luana Di Lisa [2,‡], Gianandrea Pasquinelli [1,3], Maria Letizia Focarete [2], Sabrina Valente [1,3,*]

[1] Department of Medical and Surgical Sciences, University of Bologna, 40138 Bologna, Italy; carmen.ciavarella2@unibo.it (C.C.); gianandr.pasquinelli@unibo.it (G.P.); sabrina.valente2@unibo.it (S.V.)
[2] Department of Chemistry "Giacomo Ciamician", University of Bologna, 40126 Bologna, Italy; luana.dilisa2@unibo.it (L.D.L.); marialetizia.focarete@unibo.it (M.L.F.)
[3] IRCCS Azienda Ospedaliero-Universitaria di Bologna, 40138 Bologna, Italy.

‡ These authors contributed equally to this work and share first authorship.

* Correspondence: sabrina.valente2@unibo.it

Academic Editor: Firstname Lastname

Received: date
Revised: date
Accepted: date
Published: date

Citation: To be added by editorial staff during production.

Copyright: © 2025 by the authors. Submitted for possible open access publication under the terms and conditions of the Creative Commons Attribution (CC BY) license (https://creativecommons.org/licenses/by/4.0/).

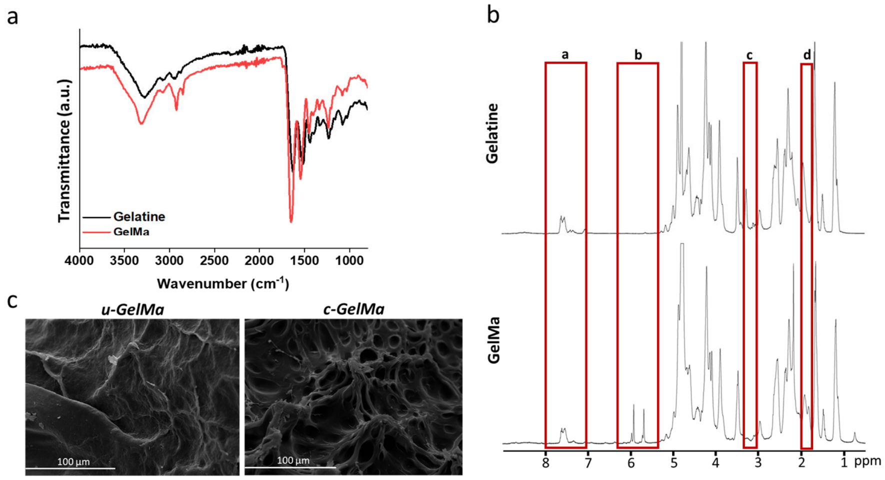

Supplementary Figure S1. a) FTIR spectra of gelatine (orange) and GelMa (red) in the range of 4000 – 800 cm^{-1}; **b)** Representative ^{1}H-NMR spectra of gelatine and GelMa. (a) Phenylalanine's aromatic rings (5 protons); (b) methacryloyl groups in GelMa as a double peak between 5.3 and 6.8 ppm absent in gelatine; (c) Arginine peak before and after methacrylation; (d) NH_2 group substitutions by methyl protons of methacryloyl groups absent in gelatine at 1.8 ppm. **c)** SEM images of hydrogel samples: uncrosslinked (u-GelMa) and crosslinked GelMa (c-GelMa).

Table S1. Summary of the elastic (G') and loss (G") moduli, crossover point for hydrogel samples at 25°C (*Amplitude Sweep Test*)

 https://doi.org/10.3390/biom15060865

Sample	G' (Pa)	G" (Pa)	Crossover Point (%)
**u-GelMa**	445	24	800
**c-GelMa**	1703	85	300

Table S2. Summary of the elastic (G') and loss (G") moduli, crossover point for c-GelMa at 37°C (*Amplitude Sweep Test*)

Sample	Condition	Time Point	G' (Pa)	G" (Pa)
**c-GelMa**	Static	0h	1791	113
**c-GelMa**	Static	24h	1023	62
**c-GelMa**	Static	48h	592	17
**c-GelMa**	Dynamic	0h	1791	113
**c-GelMa**	Dynamic	24h	256	15
**c-GelMa**	Dynamic	48h	110	11

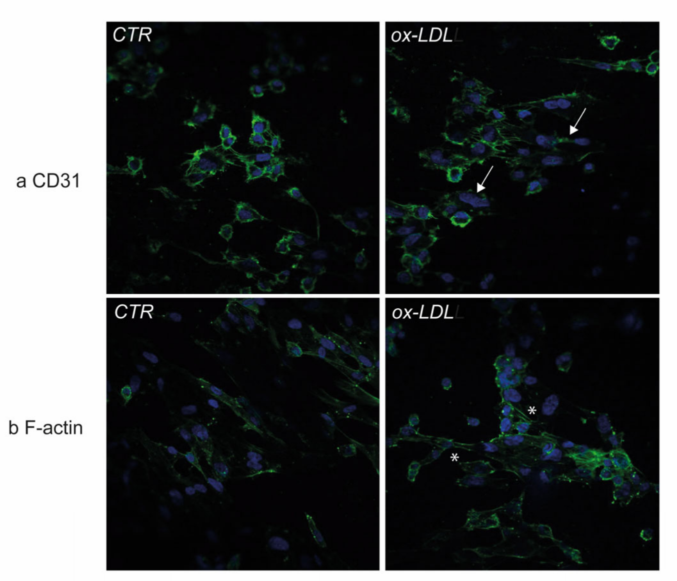

Supplementary Figure S2: CD31 expression and remodeling of F-actin filaments of HUVEC in the 3D model of vascular intima tunica. Representative images of HUVEC CTR and exposed to ox-LDL morphology; **a**) some cells showed a reduced expression for CD31 (arrows); **b**) some cells underwent a cytoskeletal remodeling of F-actin acquiring an elongated morphology (asterisks). HUVEC CTR: cells cultured in endothelial medium; HUVEC ox-LDL: cells treated with ox-LDL added to the culture medium. CD31 and F-actin images were taken to 40× magnification, respectively. Blue: nuclei counterstained with DAPI; green: HUVEC cells positive to CD31 and F-actin, respectively.

Disclaimer/Publisher's Note: The statements, opinions and data contained in all publications are solely those of the individual author(s) and contributor(s) and not of MDPI and/or the editor(s). MDPI and/or the editor(s) disclaim responsibility for any injury to people or property resulting from any ideas, methods, instructions or products referred to in the content.
