# Supplementary figures and images for: A 3D Composite Model Using Electrospinning Technology to Study Endothelial Damage
Source: Biomolecules. 2025 Jun 13;15(6):865. doi: 10.3390/biom15060865 (PMC12190949; doi:10.3390/biom15060865)

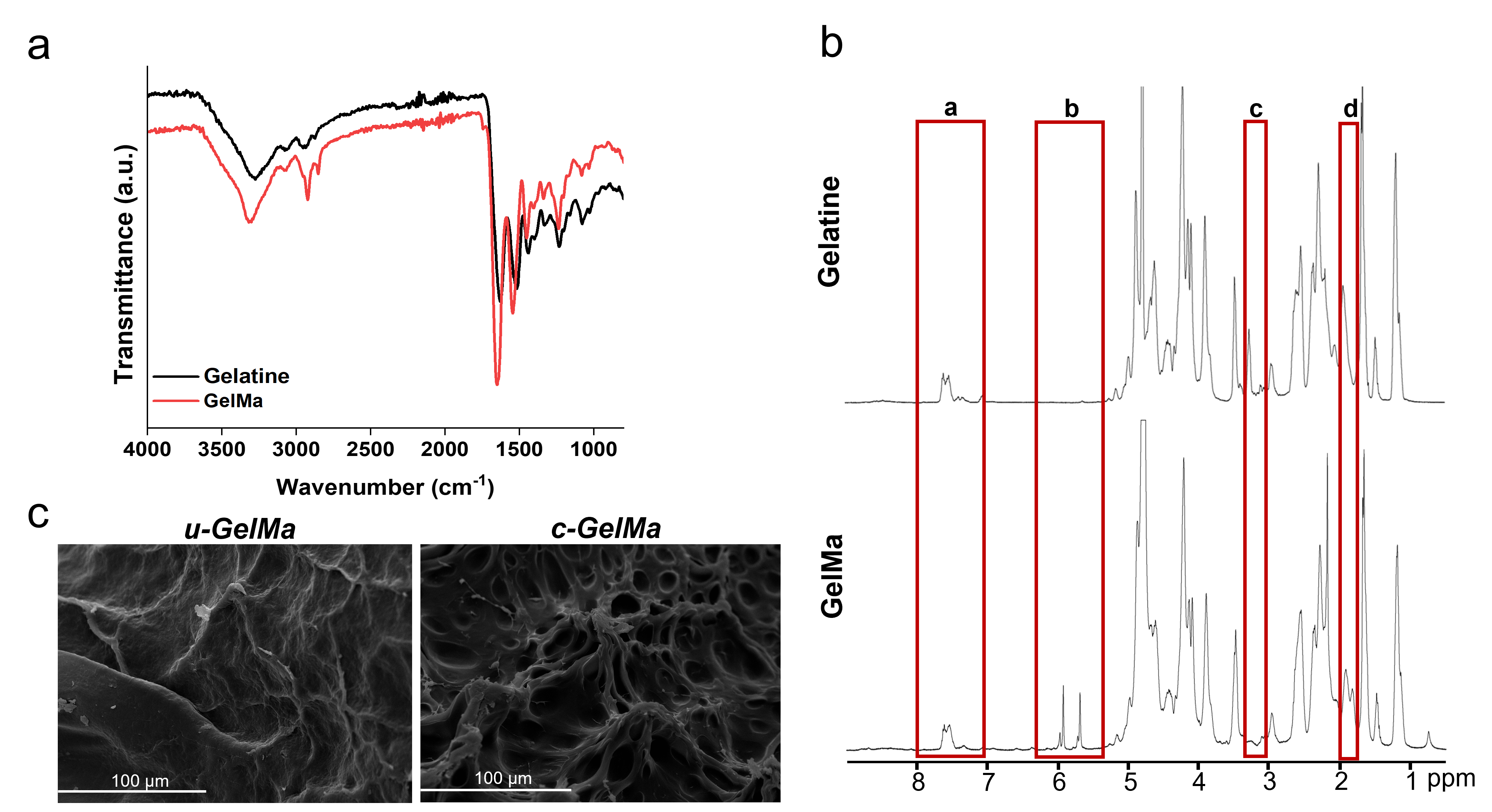

Supplement: Supplementary file 1 [file biomolecules-15-00865-s001.zip › Supplementary Figure S1_Ciavarella C et al.tif]

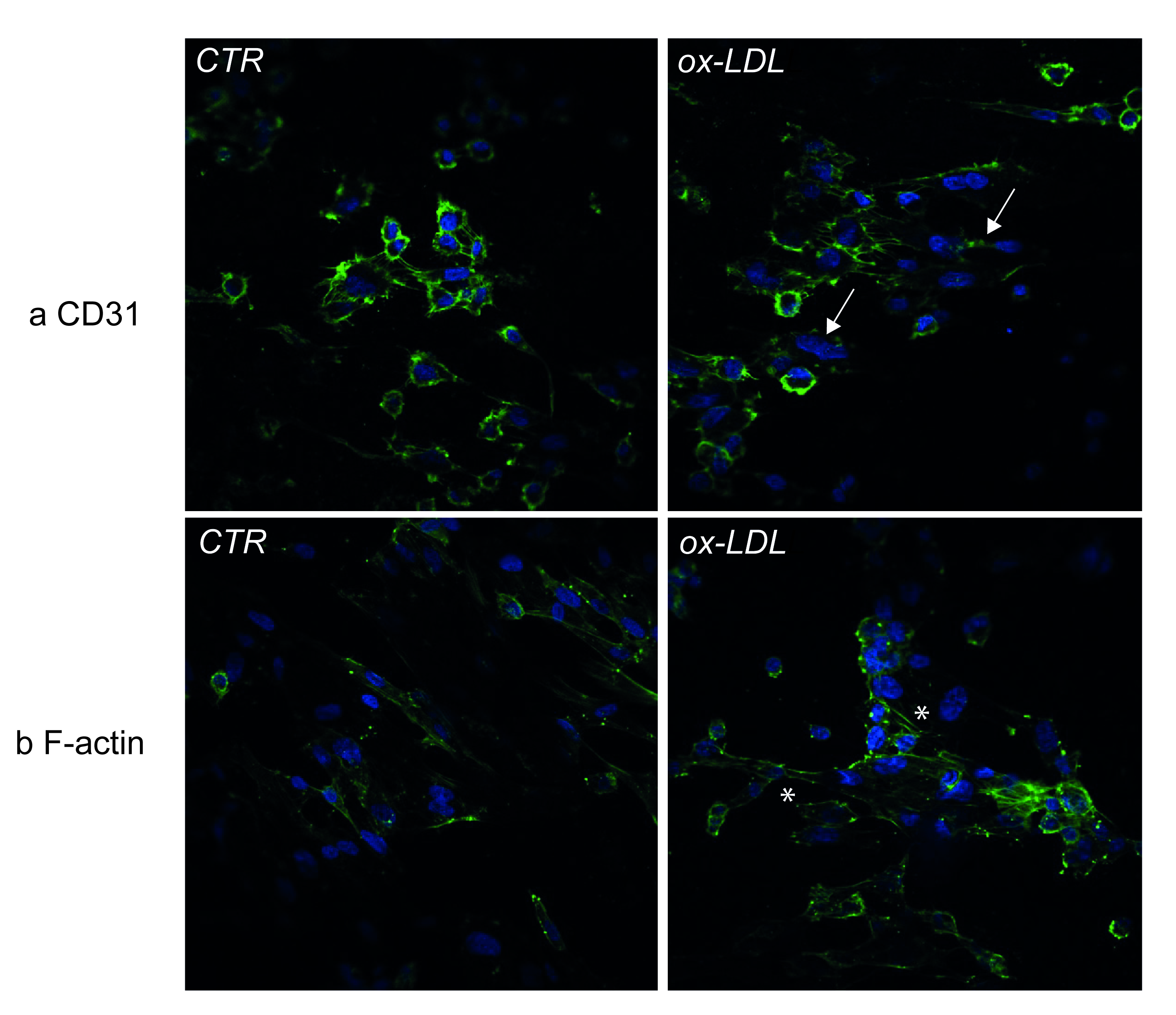

Supplement: Supplementary file 1 [file biomolecules-15-00865-s001.zip › Supplementary Figure S2_Ciavarella C et al.tif]
